# A multicentric observational trial of pegylated liposomal doxorubicin for metastatic breast cancer

**DOI:** 10.1186/1471-2407-10-2

**Published:** 2010-01-05

**Authors:** Jens Huober, Werner Fett, Arnd Nusch, Michael Neise, Marcus Schmidt, Arthur Wischnik, Steffen Gerhardt, Thomas Goehler, Hans-Joachim Lück, Andreas Rost

**Affiliations:** 1 Breast Center Kantonsspital, St Gallen, Switzerland; 2 Oncological Practice, Wuppertal, Germany; 3 Oncological Practice, Velbert, Germany; 4 Oncological Practice, Krefeld, Germany; 5 University of Mainz, Dept of Gynecology, Mainz, Germany; 6 Clinical Center Augsburg, Augsburg, Germany; 7 Oncological Practice, Gera, Germany; 8 Oncological Practice, Dresden, Germany; 9 Hannover Medical School, Hannover, Germany; 10 Clinical Center Darmstadt, Darmstadt, Germany

## Abstract

**Background:**

Pegylated liposomal doxorubicin (PLD) is active in metastatic breast cancer. This observational study evaluated the efficacy and safety of PLD in patients treated during routine clinical practice.

**Methods:**

Eligible patients had metastatic breast cancer and were treated with PLD according to the dose and schedule determined by their physician as part of routine practice. The primary objectives were to analyze the efficacy and toxicity of PLD therapy.

**Results:**

125 patients were assessable. Median age was 62 years, 78% had performance status 0-1, and 60% had estrogen-receptor-positive disease. PLD treatment was second- or third-line in 69% of patients. Prior anthracyclines (adjuvant or metastatic) had been used in 56% of patients. The majority of patients (79%) received PLD every 4 weeks at a median dose of 40 mg/m^2^. Overall response rate was 43% in all patients and 34% in those previously treated with anthracyclines. The most common grade 3/4 adverse events were skin toxicity/hand-foot syndrome (6%), and leukopenia (3%).

**Conclusions:**

This observational study supports the activity and tolerability of PLD in metastatic breast cancer as demonstrated in PLD clinical trials.

## Background

Anthracyclines are highly active as single agents in the treatment of metastatic breast cancer; however, their use is limited by acute toxicities and the potential for cumulative cardiac damage. The concern for cardiotoxicity is heightened in patients who have already received an anthracycline in the adjuvant setting [1].

The development of liposomal anthracyclines has resulted in an improved safety profile and comparable efficacy to conventional anthracyclines. In a phase III trial comparing pegylated liposomal doxorubicin (PLD) to conventional doxorubicin as first-line treatment for metastatic breast cancer (MBC), no significant difference occurred between arms in progression-free or overall survival [2]. However, cardiotoxicity was significantly reduced with PLD, along with alopecia, nausea/vomiting, and neutropenia. Hand-foot syndrome (HFS), stomatitis, and mucositis were increased with PLD. Clinical trials suggest these toxicities are dose-dependent [3,4].

Additional clinical trials have demonstrated the activity of PLD in patients previously treated with anthracyclines as well as taxane-refractory patients [5,6]. PLD is also an effective, well tolerated treatment option in elderly patients with MBC [7-9].

Due to strict eligibility requirements for enrollment, results of clinical trials are often difficult to apply to patients in everyday clinical practice. This is especially true after regulatory approval, where the doses of new oncology drugs used in daily practice often differ from the doses recommended in their prescribing information [10]. An observational study provides a useful tool for describing everyday clinical practice since in these trials the safety and efficacy of a drug in a non-selected group of patients from community practice is reported, providing evidence to support clinical trial findings. The purpose of this observational study was to determine the activity and safety of PLD as applied in routine clinical practice in Germany. Further, the applied dose and treatment schedule of PLD compared to a preselected study population was investigated.

## Methods

Patients were selected from medical practices and hospitals in 34 German cities between July, 2003 and December, 2005 for this open-label, non-randomized, multi-center observational study. Eligible patients were those who had histologically confirmed metastatic breast cancer, and for whom the decision to treat with PLD had already been made by their treating physician based on routine clinical practice as described in national and international guidelines for the treatment of MBC [11,12]. No other inclusion or exclusion criteria were applied. Due to the observational character of this study, ethical approval was not required according to the German medicines law (Arneimittelgesetz, AMG) at the time of the trial. The study was conducted in accordance with the Declaration of Helsinki and Good Clinical Practice Guidelines.

### Treatment

While the recommended dose of PLD in the prescribing information, is 50 mg/m^2 ^administered as a 30-60 minute intravenous infusion on day 1, repeated every 28 days, most practitioners use a PLD dose of 40 mg/m^2 ^[13]. A maximum of 12 cycles of PLD could be documented per patient. All aspects of treatment (eg, dose, schedule) remained at the discretion of the treating physician. However, physicians were to follow the contraindications, warnings, and precautions as stated in the product information for PLD. Treatment compliance was assessed by analyzing the documented data on dose and schedule of PLD administration. Except for progressive disease or unacceptable toxicity, no criteria were set for removal of patients from treatment or study; this decision was left to the physician's discretion. Patient characteristics, supportive therapy, preceding treatments, dose changes, and delays were documented.

### Response and Toxicity Assessment

The primary objectives were to analyze the efficacy and toxicity of PLD therapy in this unselected population. Secondary study objectives included the dose, number of cycles, and treatment schedule of PLD administered,

Efficacy, defined as complete remission, partial remission, stable disease, or disease progression, was assessed by the evaluation of clinical response parameters according to best clinical practice [14]. Response was evaluated as "best response to therapy" once per patient. Occurrence of progressive disease was also documented. Median time of progression-free and overall survival were estimated by Kaplan-Meier analysis. Toxicities were classified according to the National Cancer Institute Common Terminology Criteria for Adverse Events (CTCAE) in the version 2.0 of 1994. Adverse events were recorded at the end of every cycle.

### Statistical Analysis

Data from patients who received at least one cycle of PLD and whose documentations were completed were analyzed for toxicity and efficacy. Descriptive and explorative statistical analysis was performed for patient baseline data, therapy data, and response data. Kaplan-Meier analysis was used to evaluate progression-free and overall survival.

## Results

Data from 132 women were evaluated for inclusion in the analysis. Of these, 125 patients who received at least one cycle of PLD and whose documentations were completed, checked and signed by the physician were assessable for analysis of efficacy and toxicity. Patient demographics and tumor characteristics are detailed in Table 1. The majority (n = 123) of patients had received prior hormonal therapy or chemotherapy. PLD was administered as first-line therapy in 14 patients (11%) and as second- or third-line therapy in 86 patients (69%). Over half (n = 70; 56%) had received prior anthracycline-based chemotherapy either in the adjuvant or metastatic setting.

**Table 1:** Patient Demographics and Tumor Characteristics

Characteristic	No. Patients (%) N = 125
**Median age, years (range)**	62 (37-84)
**Performance status (ECOG)**	
0-1	98 (78)
2	21 (17)
3	3 (2)
Unknown	3 (2)
**Hormone receptor status**	
Positive	75 (60)
Negative	37 (30)
Unknown	13 (10)
**Active Treatment setting**	
Neoadjuvant	1 (<1)
Adjuvant	1 (<1)
First-line advanced	14 (11)
Second-line advanced	51 (41)
Third-line advanced	35 (28)
Fourth-line advanced or greater	23 (18)
**Prior treatments**	
Surgery	116 (93)
Hormone therapy	89 (71)
Prior anthracycline therapy	70 (56)
Palliative chemotherapy	81 (65)
Adjuvant chemotherapy	54 (43)
Immunotherapy	8 (6)
**Site of metastasis**	**N = 123**
Bone	69 (56)
Liver	56 (4)
Lung	44 (36)
Skin	14 (11)
Lymph nodes	13 (11)
Pleura	8 (6)
Brain	7 (6)
Others	26 (21)

For all patients, 649 cycles of PLD were recorded with a median of 4 cycles per patient (range, 1 to 12). The most common duration of therapy was 4 courses received by 27 patients (21%), followed by 6 courses (25 patients, 20%) and 3 courses (24 patients, 19%). The maximum number of 12 cycles was reported for 7% of patients.

Treatment intervals were at the physician's discretion. Schedules of every 2, 3, 4, or 5 weeks were reported, with the median PLD dose dependent on the schedule. Patients generally received approximately 10 mg/m^2 ^per week (Table 2). Administration every 4 weeks was the most frequently-used interval, applied in 79% of the patients.

**Table 2:** Treatment Schedule and Dose

Treatment Interval (Cycle Length)	No. Patients (%) N = 120*	Median PLD Dose (mg/m^2^)	PLD Dose Range (mg/m^2^)
2 weeks	7 (6)	20	15-20
3 weeks	8 (7)	32	27-40
4 weeks	95 (79)	40	20-50
5 weeks	10 (8)	40	30-50

* Five patients had only one documented cycle of PLD and therefore treatment interval could not be analyzed.

Dose reductions occurred in 27 patients (22%), and 38 of the 649 courses (6%); non-hematologic toxicities were the most frequent cause. Dose delays occurred in 37 patients (30%), which was 47 (7%) of courses. Patient wish was the most frequently cited reason for treatment delay (19 of the 47 courses). Toxicity, either hematologic or non-hematologic, led to delay in less than 2% of treatment courses. Because a maximal number of courses was not defined, discontinuation of therapy was left to the physician's discretion and could include more than one reason. The primary reasons for treatment discontinuation included progressive disease (39% of patients) and end of regular therapy (36% of patients).

### Efficacy

Response rates in all patients and in those with prior anthracycline exposure are detailed in Table 3. The overall response rate was 42.4% in all patients and 34.3% in those previously treated with anthracyclines. Considering stable disease, a benefit was seen in 72% of all patients and in 70% in those previously treated with anthracyclines. When broken down by line of treatment, response rates for patients in the first- and second-line treatment setting was 47.8%; patients with > 2 lines of treatment had a response rate of 36.2% (Table 3).

**Table 3:** Best Response to PLD Therapy

Best Response	All Patients N = 125	Patients with Prior Anthracycline Therapy N = 70	Response by Line of Treatment	
			1^st^/2^nd ^Line	≥ 3^rd ^Line
			N = 65	N = 58
	No. Patients (%)			
Complete response	6 (4.8)	2 (2.9)	4 (6.2)	2 (3.4)
Partial response	47 (37.6)	22 (31.4)	27 (41.5)	19 (32.8)
Stable disease	37 (29.6)	25 (35.7)	15 (23.1)	22 (37.9)
Progressive disease	28 (22.4)	17 (24.3)	18 (27.7)	10 (17.2)
Not determined	7 (5.6)	4 (5.7)	1 (1.5)	5(8.6)

**Figure 1:**
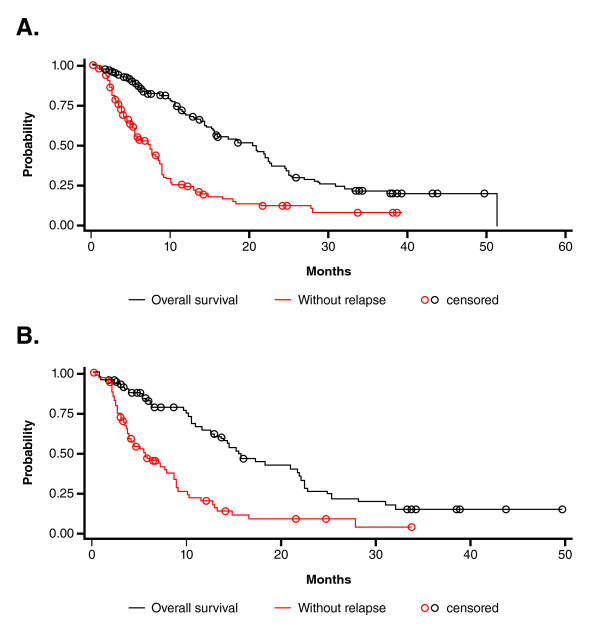
**Overall and Progression-Free Survival**. **(A) **Kaplan-Meier estimates of overall survival and progression-free survival in patients treated with PLD for metastatic breast cancer. **(B) **Kaplan-Meier estimates of overall survival and progression-free survival in anthracycline-pretreated patients treated with PLD for metastatic breast cancer.

Kaplan-Meier estimates of progression-free and overall survival in the overall population are shown in Figure 1A. The median progression-free survival time was 7.2 months (95% CI: 5.4-8.6), and the median overall survival time was 20.8 months (95% CI: 15.4-22.6). In the 70 patients pretreated with anthracycline chemotherapy, the median progression-free survival time was 5.5 months (95% CI: 3.8-8.6), and the median overall survival time was 15.4 months (95% CI: 12.5-21.9; Figure 1B).

### Safety

No unexpected toxicities occurred. The most common adverse events of all grades were anemia, leukopenia, alopecia, skin toxicity, pain, stomatitis, and nausea (Table 4). The most frequent grade 3 or 4 adverse events were skin toxicity/hand-foot syndrome (6%), and leukopenia (3%). Grade 2 skin toxicity/hand-foot syndrome, which can also be bothersome, was seen in 14% of patients. Five cardiac adverse events were reported in 4 patients, in all but 1 recorded as grade 1 or 2 in severity: cardiac arrhythmia and heart palpitation (in the same patient), heart palpitation, left ventricular systolic dysfunction (in one patient grade 3). Death was documented for 9 patients. In one case, the death was recorded as possibly related to PLD treatment. Of the remaining 8 deaths, 6 were considered by the physician to be not related to treatment (due to progressive disease) and 2 occurred greater than 3 months after the last PLD cycle and were therefore not reported as adverse events.

**Table 4:** Adverse Events

Toxicity	Grade 1	Grade 2	Grade 3	Grade 4	Grade Not Determined
	No. Patients (%) N = 125				
**Hematologic**					
Anemia	31 (25)	12 (10)	1 (<1)	0	6 (5)
Leukopenia	24 (19)	8 (6)	3 (2)	1 (<1)	6 (5)
Neutropenia	12 (10)	0	0	0	19 (15)
Thrombocytopenia	14 (11)	4 (3)	1 (<1)	1(<1)	6 (5)
**Non-hematologic**					
Skin/hand-foot syndrome	20 (16)	17 (14)	7 (6)	1 (<1)	2 (2)
Pain	22 (18)	8 (6)	4 (3)	0	4 (3)
Alopecia	13 (10)	6 (5)	-	-	7 (6)
Nausea	25 (20)	4 (3)	0	0	3 (2)
Stomatitis	16 (13)	6 (5)	2 (2)	0	5 (4)
Vomiting	5 (4)	2 (2)	1 (<1)	0	4 (3)
Diarrhea	3 (2)	3 (2)	0	0	5 (4)
Infection	0	1 (<1)	0	0	0
Fatigue	0	0	1 (<1)	0	0

## Discussion

From this observational study, practice patterns surrounding the use of PLD as well as the activity and safety of PLD as applied in routine clinical practice for patients with metastatic breast cancer can be characterized. The majority of patients received PLD as second- or third-line therapy; only 11% received this agent as first-line treatment. While a variety of dosing schedules were employed, nearly 80% received their PLD dose every 4 weeks (usually 40 mg/m^2^) and about two-thirds of these received 4 to 6 cycles. While the recommended dose of PLD in the prescribing information is 50 mg/m^2 ^repeated every 28 days, most practitioners use a PLD dose of 40 mg/m^2 ^throughout palliative settings most likely because of higher tolerability [3,15]. Thus PLD doses of 50 mg/m^2 ^every 28 days as usually administered in clinical trials is not widely used in routine clinical practice.

The activity of PLD in metastatic breast cancer in different treatment lines as monotherapy or in combination therapy has been demonstrated in multiple phase II and III studies [2,5,6,8,16,17]. In a randomized phase III trial, PLD was demonstrated to be equally effective to conventional doxorubicin as first-line treatment of women with MBC [2]. The primary advantage of PLD in this and other studies was its favorable toxicity pattern, with low hematologic toxicity, a low rate of alopecia, less nausea and vomiting, and a diminished risk of cardiotoxicity when compared to conventional doxorubicin.

Though a limitation of this study is its observational nature with heterogeneous treatment schemes and different nonuniform toxicity management practices, the study supports the activity, tolerability and safety of PLD in an unselected population treated in routine clinical practice. In this study, no specifications with respect to tumor measurement or response confirmation were made as would be present in the conduct of a clinical trial. Therefore, limited comparisons can be made with published clinical trials. The majority of patients in this study (87%) received PLD as second-line or later therapy. Complete or partial response was documented in 53 patients (42.4%), with a clinical benefit rate of 72%. Response rates observed in published clinical trials of PLD in previously-treated patients with metastatic breast cancer (second-line or higher) have ranged from 10% to 31% [5,6,16]. The higher response rates in our study could be due to the response criteria not being as strict as those in a clinical trial. Furthermore, it is not uncommon for response rates to be lower when assessed by independent review as compared to investigator assessment. Nevertheless, a clinical benefit with PLD was apparent in this study.

The mostly used dose of PLD in this observational study was 40 mg/m^2^. Even though no direct comparisons between a PLD dose of 40 mg/m^2 ^and 50 mg/m^2 ^exist, indirect comparisons suggest that 40 mg/m^2 ^is equally effective to 50 mg/m^2 ^with distinctively lower side effects, especially skin toxicity [3,18,19].

Several trials have demonstrated that PLD is active in patients previously treated with anthracyclines, as well as in patients progressing on anthracyclines, suggesting incomplete cross-resistance between PLD and conventional anthracyclines [5,6,20]. More than half of the patients in our study (N = 70) had been previously treated with an anthracycline, and attained a 34.3% response rate and a 70% clinical benefit rate to PLD, corroborating as well a benefit of PLD in an anthracycline pretreated patient population.

Our investigation showed that PLD could be safely administered in clinical practice, with a low rate of severe toxicities. Specifically, grade 3 and 4 hematologic and gastrointestinal toxicities were very rare. Furthermore, complete alopecia was only seen in very few patients (5%). In the palliative setting with limited life expectancy, this is a major advantage compared to doxorubicin. The most frequent PLD-associated side effects in clinical practice, such as skin toxicity/hand-foot syndrome and mucositis, were similar to those reported in clinical trials. Of note, grade 3 and 4 events were only observed in 7% and 2% of patients, respectively. In randomized phase III trials, the incidence of grade 3/4 hand-foot syndrome has been 17%-19% [2,6]. The lower incidence of hand-foot-syndrome in our study may be due to the freedom physicians had to administer lower doses of PLD and to use individual treatment schedules. Also, while randomized phase III trials utilized a PLD dose of 50 mg/m^2 ^every 4 weeks, patients in this study received median PLD doses of 40 mg/m^2 ^every 4 weeks so that patients generally received 10 mg/m^2 ^PLD per week, a dose slightly lower than the recommended 12.5 mg/m^2 ^per week (equivalent to 50 mg/m^2^/4 weeks). Indeed, reductions in hand-foot syndrome have been observed in phase II trials utilizing doses similar to what was used in this study. For example Coleman et al observed a 2% rate of grade 3-4 hand-foot syndrome events with a PLD dose of 60 mg/m^2 ^every 6 weeks, and Al-Batran et al observed no grade 3/4 HFS with a PLD dose of 40 mg/m^2 ^every 4 weeks (each dosing schedule equivalent to approximately 10 mg/m^2^/week) [3,4]. Salzberg et al observed grade 4 HFS only in patients treated with 50 mg/m^2^[21]. It should also be noted that the frequency of examinations are not defined in an observational study; therefore, patients may not be queried about adverse events as frequently as would occur during the conduct of an interventional clinical trial.

## Conclusions

In conclusion, this observational study, evaluating the use of PLD for the treatment of MBC in routine clinical practice, supports the activity and tolerability of PLD as demonstrated in clinical trials. Even in heavily pretreated patients PLD could be safely administered with low severe hematologic and non-hematologic toxicity. The doses and schedules utilized in clinical practice are slightly lower than recommended in the prescribing information and appear to be active while reducing the incidence of hand-foot syndrome.

## Competing interests

JH honoraria Essex.

## Authors' contributions

JH, MS, HJL conceived of the study; WF, AN, MN, MS, AW, SG, TG, AR provided patients for the study; JH provided data analysis, interpretation and manuscript writing; JH, WF, AN, MN, MS, AW, SG, TG, HJL, and AR provided final review and approval of the manuscript. All authors read and approved the final paper.

## Pre-publication history

The pre-publication history for this paper can be accessed here:

http://www.biomedcentral.com/1471-2407/10/2/prepub
